# Clinical laboratory reference values amongst children aged 4 weeks to 17 months in Kilifi, Kenya: A cross sectional observational study

**DOI:** 10.1371/journal.pone.0177382

**Published:** 2017-05-11

**Authors:** Jesse Gitaka, Caroline Ogwang, Moses Ngari, Pauline Akoo, Ally Olotu, Christine Kerubo, Greg Fegan, Patricia Njuguna, Godfrey Nyakaya, Tuda Otieno, Gabriel Mwambingu, Ken Awuondo, Brett Lowe, Roma Chilengi, James A. Berkley

**Affiliations:** 1 Clinical Trials Facility, Kenya Medical Research Institute/Wellcome Trust Research Programme, Kilifi, Kenya; 2 Department of Clinical Medicine, School of Health Sciences, Mount Kenya University, Thika, Kenya; 3 The Childhood Acute Illness & Nutrition Network (CHAIN), Nairobi, Kenya; 4 Centre for Tropical Medicine & Global Health, University of Oxford, Oxford, United Kingdom; 5 Swansea Trials Unit, Swansea University Medical School, Swansea University, Swansea, United Kingdom; 6 Centre for Infectious Disease Research in Zambia, Lusaka, Zambia; Food and Drug Administration, UNITED STATES

## Abstract

Reference intervals for clinical laboratory parameters are important for assessing eligibility, toxicity grading and management of adverse events in clinical trials. Nonetheless, haematological and biochemical parameters used for clinical trials in sub-Saharan Africa are typically derived from industrialized countries, or from WHO references that are not region-specific. We set out to establish community reference values for haematological and biochemical parameters amongst children aged 4 weeks to 17 months in Kilifi, Kenya. We conducted a cross sectional study nested within phase II and III trials of RTS, S malaria vaccine candidate. We analysed 10 haematological and 2 biochemical parameters from 1,070 and 423 community children without illness prior to experimental vaccine administration. Statistical analysis followed Clinical and Laboratory Standards Institute EP28-A3c guidelines. 95% reference ranges and their respective 90% confidence intervals were determined using non-parametric methods. Findings were compared with published ranges from Tanzania, Europe and The United States. We determined the reference ranges within the following age partitions: 4 weeks to <6 months, 6 months to less than <12 months, and 12 months to 17 months for the haematological parameters; and 4 weeks to 17 months for the biochemical parameters. There were no gender differences for all haematological and biochemical parameters in all age groups. Hb, MCV and platelets 95% reference ranges in infants largely overlapped with those from United States or Europe, except for the lower limit for Hb, Hct and platelets (lower); and upper limit for platelets (higher) and haematocrit(lower). Community norms for common haematological and biochemical parameters differ from developed countries. This reaffirms the need in clinical trials for locally derived reference values to detect deviation from what is usual in typical children in low and middle income countries.

## Introduction

In clinical trials, reference values for clinical laboratory parameters are important for screening for eligibility; diagnosis and grading of toxicities; and management of adverse events. The reference ranges and toxicity grading scales for haematological and biochemical parameters typically used for clinical trials in sub-Saharan Africa are usually derived from industrialized countries or from WHO references that are not region-specific [1–5]. However, typical laboratory parameters in communities may vary based on race, age, gender, diet, local disease patterns and environmental characteristics [6–11].

An upsurge of clinical trials in developing countries in the recent years has led to questions over the use of external references, and the need to determine what is ‘normal’ in local communities in order to be able to interpret eligibility for representative participation in trials and potential adverse events [4, 12–14]. ‘Normal’ may not be ‘optimal’, thus the target population who may ultimately receive an intervention that is being tested may have characteristics that fall outside international reference values when in their usual state of health [15, 16]. Moreover, available reference data may not adequately cover the youngest age groups, be gender-specific, or may have been determined using older instruments or when characteristics of the population and interventions such as bed net distribution or micronutrient supplementation may have been different [17–20].

Reference intervals for biological parameters are usually defined as values falling within two standard deviations (95% prediction) of the mean found in healthy populations [21]. The US-based Clinical Laboratory and Standards Institute (CLSI) guidelines recommend that laboratories establish their reference intervals for their own population, or validate those obtained from a different setting [22, 23].

Adult populations in Africa often have lower haemoglobin, red blood cell counts, haematocrit, mean corpuscular volume, platelets and neutrophils, and higher monocyte and eosinophil counts than Caucasian populations [3, 8, 24]. Creatinine and transaminases are generally comparable to that of Caucasians [16, 23, 24]. Similarly, studies in African children have typically shown that red blood cell parameters such as haemoglobin, haematocrit and mean corpuscular volume are lower than those of Caucasians, with the exception of platelets [25, 26] and eosinophils, which tend to be higher, the latter being attributed to helminth infestation.

The aim of this study was to establish age-specific haematological and biochemistry reference values for typical children aged 4 weeks to 17 months living in Kilifi County in rural Kenya who participated in malaria vaccine trials [27, 28].

## Methods

### Study site

The study took place in Kilifi district (now part of Kilifi County), Kenya, latitude -3.63, longitude 39.85 degrees [29]. It is a predominantly rural area and the majority of the inhabitants are subsistence farmers from the Mijikenda ethnic group. The staple diet is corn meal, cassava and local green vegetables. The main income generating activities are tourism, small-scale trading, farming, fishing and employment in nearby towns [30]. The climate is tropical with long rains being between April to July and short rains between October and December. There has been a downward trend of malaria prevalence locally and reduction in the densities of the major malaria vectors, with a shift from human to animal feeding [31, 32].

### Study participants

We carried out a cross sectional study nested within phase IIb [27] and phase IIIb [28] RTS,S malaria candidate vaccine trials. Further information on trial design and methodology can be found in the primary trial publications [27, 28].

A series of public meetings were held in the study communities and parents who showed interest in the study were invited to bring their children for further examination at the study dispensary. Parents/guardians gave written consent for their children to be enrolled into these trials and only children who were judged to have no serious acute or chronic illness as determined by history and physical examination (defined as not having any signs and symptoms of disease, ambulatory (children older than 1 year) and not underweight, defined as weight-for-age Z score (WAZ) ≥-2), medical history records or laboratory screening tests were eligible. Other exclusion criteria included a history of allergic reactions, a history of a previous blood transfusion, major congenital defects, or a confirmed or suspected immunodeficiency disorder (e.g. active HIV disease of Stage III or Stage IV severity, as defined by the World Health Organization, at the time of screening). There was no routine testing for HIV in the trials.

Blood samples from eligible children were then obtained before immunisation for haematology (for both trials), and creatinine and alanine transaminase (only for the phase IIb trial). We only used laboratory results taken at baseline (the screening results), prior to the administration of trial interventions which may have otherwise influenced physiological parameters. The only haematology parameter that was consistently done for all participants was haemoglobin. Children with severe anemia, defined as a haemoglobin concentration <5.0g/dL or a haemoglobin concentration <8.0g/dL associated with clinical signs of heart failure or severe respiratory distress were then excluded.

A total of 450 children aged 5–17 months were recruited between March 2007 and August 2007 in the phase IIb trial and a further 904 children were recruited 2009 to 2014 in the phase IIIb trial in two age cohorts: 5–17 months (N = 600), and 6 to 12 weeks (N = 304).

### Blood collection

Capillary and venous samples were collected from finger pricks or the antecubital fossa veins respectively. Infants more frequently had capillary sampling due to feasibility. Samples were collected in a 0.5 ml di-potassium ethylene diamine tetra acetic acid (K_2_EDTA) tube for haematology and 1ml serum separator tube (SST) tube for biochemistry. Blood samples were transported in cool boxes containing ice packs within four hours of collection to the KEMRI/Wellcome Trust Clinical Trials Laboratory. All samples were collected in the morning hours before midday and processed within 6 hours of collection.

### Laboratory analysis

Haematological parameters examined were haemoglobin (Hb), haematocrit (Hct), mean corpuscular haemoglobin concentration (MCHC)**,** mean corpuscular volume (MCV)**,** platelets, white blood count (WBC) and differentials (neutrophils, lymphocytes, monocytes, eosinophils, and basophils) using a Beckman Coulter *AcT 5 Diff* Haematology *Analyzer* (Beckman Coulter, USA) [33]. The biochemical parameters were alanine transaminase (ALT) and creatinine (Cr), analysed using a Vitalab Selectra-E clinical chemistry analyser (Vital Scientific (Merck), Netherlands).

Normal and abnormal controls were run daily and no analysis was done if controls were out of range. Assays were done according to manufacturer’s instructions and established standard operating procedures. The KEMRI/Wellcome Trust Clinical Trials Laboratory is a member of two external quality assurance schemes; United Kingdom’s National External Quality Assessment Service (since 2003) and the Royal College of Pathologist of Australasia–Quality Assurance Program (since 2006); and is also Good Clinical Laboratory Practice (GCLP) accredited by Qualogy Limited since 2006.

### Statistical methods

Data were double-entered to an OpenClinica® database from source documents. Data analysis was carried out using Stata 12 (Stata Corp, College Park, TX, USA) and all statistical tests considered significant at P<0.05 (two-sided). To obtain the reference ranges we followed the Clinical and Laboratory Standards Institute (CLSI) 2008 guidelines (EP28-A3c) [34]. CLSI recommends a minimum of 120 samples for analysis by nonparametric methods for each partition (e.g. gender, age range).

The sample population was stratified by gender and categorized into the following preconceived age groups; 4 weeks to less than 6 months, 6 months to less than 12 months and 12 months to 17 months. We computed 95% reference ranges, and the 90% confidence limits around each of the upper and lower reference values. Statistical differences by gender were assessed using a Wilcoxon rank-sum test.

For haematology and biochemical variables, we assessed and excluded outlying values in the analysis using Tukey’s method [35], as recommended by CLSI for establishing reference intervals [34, 36] where there are multiple outliers. It comprises calculating the interquartile range (IQR) for each age group, then the lower and upper boundaries were computed as follows; a) lower boundary = 25^th^ percentile– 1.5*IQR and b) upper boundary = 75^th^ percentile + 1.5*IQR. Any data points that were outside the lower and upper boundaries were considered outliers and excluded from the ranges.

For comparison with other published reference ranges from Tanzania and European/American children [26, 37], the 90% ranges for lower and upper 95% reference limit were computed for each parameter for ages 1 to 12 months. Tanzania and European/American children 95% lower and upper reference limits were considered not comparable if not within the Kenyan 90% ranges. To compare to our 95% reference ranges, we estimated the means and standard deviation using the number of participants and the 95% reference values, then used one sample t-tests[38]. We could only compare children aged < 1year because this was the age group with available published data.

### Ethical considerations

The study protocols of the original vaccine trials were approved by the Kenya Medical Research Institute Ethics Review Committee and regulatory approval was obtained from the Expert Committee on Clinical Trials of the Pharmacy and Poisons Board. The studies were conducted according to the study protocols, Declaration of Helsinki, the International Conference on Harmonization’s Good Clinical Practice standards and the Kenyan regulatory requirements. This secondary analysis was approved by the sponsor, the Malaria Vaccine Initiative (MVI), and the Kenya Medical Research Institute Ethics Review Committee.

## Results

There were baseline laboratory results available for a total of 1,294 enrolled subjects: 423 (33%) from the phase IIb trial and 871 (67%) from the phase III trial. Data from 61 (4.7%) participants were excluded because they were outside the target age range. A further 163 (13%) were excluded because they were underweight (WAZ <-2). Among the 1070 participants included in analysis, 450(42%) were female, the median age was 8.2 (IQR 3.2–12.5) months, the median WAZ was -0.54 (IQR -1.18 to 0.12).

**Fig 1:**
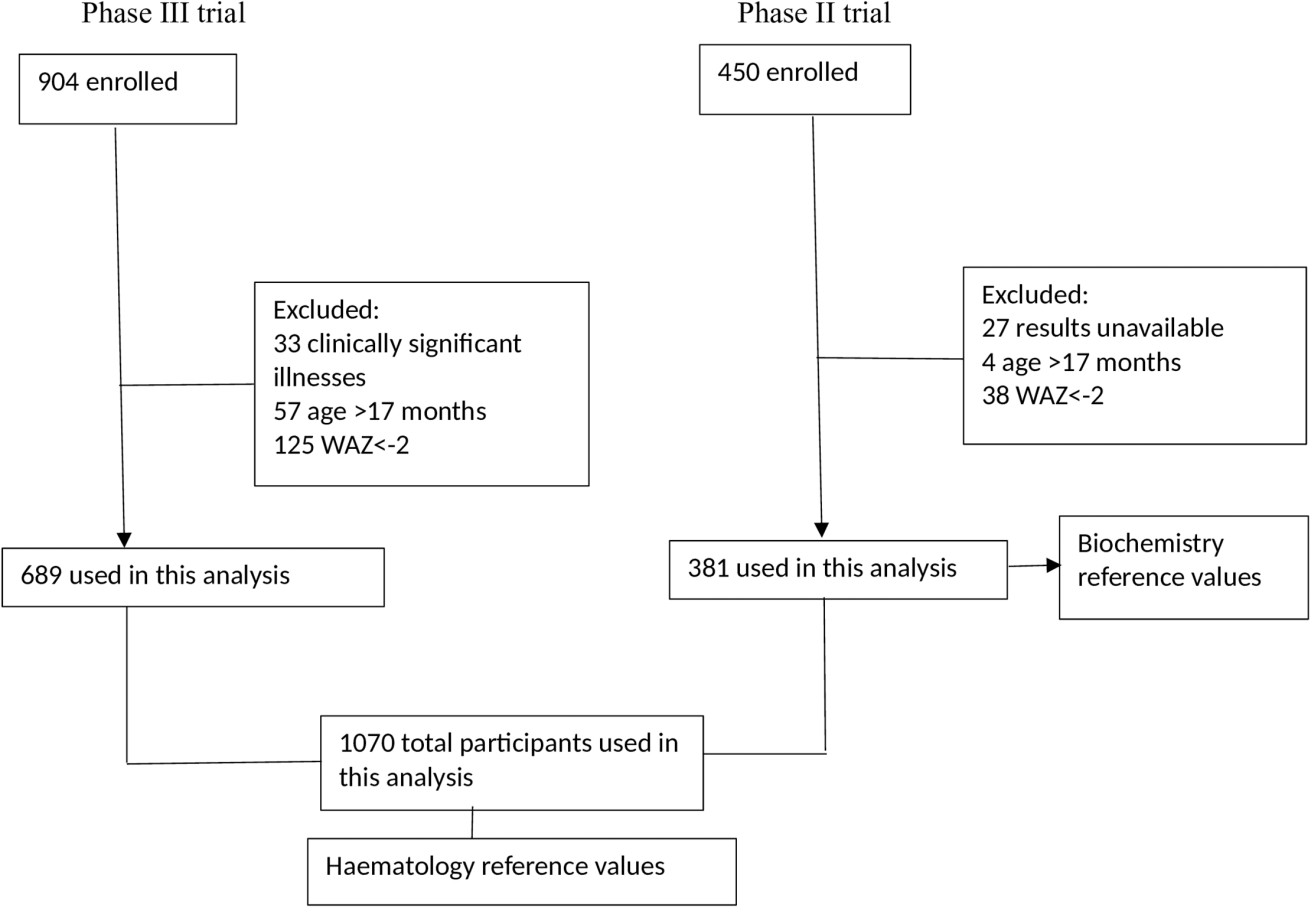
Participant flow diagram.

For haematology values, results from 1,070 children from both trials were included. Biochemical values were only obtained during the phase IIb RTS,S trial and 381 results were analysed (Fig 1).

The 95% reference ranges and the 90% confidence intervals for the upper and lower limits for the red blood cell indices and platelet counts; white blood cell and chemistry parameters are displayed in Tables 1, 2 and 3 respectively. The 95% reference ranges for creatinine and ALT for 4 weeks to 17 month old children were 26.5 to 45.0 μmol/L and 9.0 to 34.0 IU/L respectively (Table 3).

**Table 1:** 95% reference ranges with 90% confidence intervals for selected haematological parameters for Kilifi children aged 1–17 months.

Parameter/Age group	N*	95% Reference range	90% Confidence interval (Lower reference limits)	90% Confidence interval (Upper reference limits)
**Haemoglobin (g ⁄ dl)**				
1 to 6 months	358	8.1–13.8	7.6–8.4	13.6–14.1
6 to 12 months	386	7.0–11.5	6.9–7.3	11.4–11.8
12 to 17 months	306	7.1–11.9	6.9–7.5	11.6–12.1
Overall	1050	7.2–12.7	7.0–7.5	12.6–12.9
**Haematocrit (%)**				
1 to 6 months	299	24.8–41.9	23.8–25.7	40.9–43.2
6 to 12 months	321	23.2–36.1	22.6–23.8	35.1–36.7
12 to 17 months	222	25.0–36.6	23.4–25.6	35.7–37.1
Overall	842	23.9–38.3	23.3–24.7	37.8–38.7
**MCHC (g/dL)**				
1 to 6 months	301	31.2–34.7	30.9–31.4	34.5–35.1
6 to 12 months	322	29.5–33.8	29.2–29.9	33.7–34.1
12 to 17 months	224	28.9–33.5	28.2–29.0	33.5–34.2
Overall	847	29.4–34.4	29.2–29.6	34.2–34.5
**MCV (fl)**				
1 to 6 months	305	57–100	55–59	98–103
6 to 12 months	328	51–78	50–53	77–80
12 to 17 months	227	50–79	48–51	77–81
Overall	860	52–97	51–53	96–98
**Platelets (×10**^**3**^ **cells/**μ**L)**				
1 to 6 months	303	74–765	23–113	713–835
6 to 12 months	318	104–798	84–151	756–837
12 to 17 months	217	184–769	142–220	747–807
Overall	838	84–773	55–100	752–812

N* varies for each age group as some lab tests were not done for all participants and the following outliers were excluded from analysis:Hb-19, Hct-5, MCHC-8, MCV-0, Platelets- 15.

**Table 2:** 95% reference ranges with 90% confidence intervals for selected white blood cells parameters for Kilifi children aged 1–17 months.

Parameter/Age group	N*	95% reference values	90% confidence interval (Lower reference Limits)	90% confidence interval (Upper reference Limits)
**WBC(×10**^**3**^ **cells/**μ**L)**				
1 to 6 months	293	4.74–14.77	4.50–5.41	13.75–15.87
6 to 12 months	321	6.70–17.39	6.20–7.04	16.70–17.91
12 to 17 months	218	5.84–16.66	5.05–6.50	16.10–17.75
Overall	832	5.71–16.72	5.31–6.05	16.41–17.08
**Neutrophils(×10**^**3**^ **cells/**μ**L)**				
1 to 6 months	211	0.57–3.53	0.37–0.68	3.32–3.77
6 to 12 months	234	1.05–4.25	0.71–1.12	3.93–4.39
12 to 17 months	137	0.95–5.56	0.76–1.02	4.89–5.97
Overall	582	0.70–4.39	0.64–0.82	4.18–4.45
**Lymphocytes(×10**^**3**^ **cells/**μ**L)**				
1 to 6 months	229	3.06–9.04	2.14–3.45	8.52–9.47
6 to 12 months	237	3.38–10.97	3.06–3.71	10.83–11.35
12 to 17 months	137	2.97–9.75	2.19–3.21	9.38–10.37
Overall	603	3.13–10.20	2.99–3.39	9.87–10.77
**Monocytes(×10**^**3**^ **cells/**μ**L)**				
1 to 6 months	218	0.38–1.89	0.28–0.44	1.81–1.98
6 to 12 months	234	0.60–2.06	0.51–0.65	1.96–2.17
12 to 17 months	134	0.48–1.91	0.31–0.55	1.72–1.94
Overall	586	0.48–1.93	0.43–0.52	1.91–2.01
**Eosinophils(×10**^**3**^ **cells/**μ**L)**				
1 to 6 months	211	0.07–0.70	0.05–0.11	0.61–0.78
6 to 12 months	232	0.07–1.20	0.04–0.09	1.11–1.31
12 to 17 months	134	0.05–1.20	0.03–0.08	0.99–1.28
Overall	577	0.06–0.98	0.05–0.08	0.92–1.02
**Basophils(×10**^**3**^ **cells/**μ**L)**				
1 to 6 months	193	0.01–0.06	0.00–0.01	0.05–0.07
6 to 12 months	214	0.01–0.04	0.00–0.01	0.04–0.04
12 to 17 months	123	0.01–0.06	0.00–0.01	0.04–0.06
Overall	583	0.01–0.06	0.00–0.01	0.05–0.06

N* varies for each age group as some lab tests were not done for all participants and the following outliers were excluded from analysis: (WBC– 29, Neutrophils- 31, Lymphocytes- 21, Monocytes- 27, Eosinophils- 36, Basophils- 30).

**Table 3:** 95% reference ranges with 90% confidence intervals for selected biochemistry parameters for Kilifi children aged 1–17 months.

Parameter	N*	95% Reference values	90% Confidence interval (Lower reference Limits)	90% Confidence interval (Upper reference Limits)
**Creatinine (μmol/L)**	419	27–45	25–28	44–46
**ALT (IU/L)**	408	9–34	7–10	32–35

N* varies for each parameter because the following outliers were excluded from analysis: ALT- 14, Cr-3.

There were no statistically significant differences by gender for all assessed haematological and biochemical parameters (S1 Table, S2 Table and S3 Table).

Reference haematological values for Tanzanian and European/American infants were examined for comparison (Tables 4 and 5 and S4 Table). We did not find appropriate published reference intervals covering 4 weeks to 17 months of age to compare with our biochemical parameters.

**Table 4:** 95% reference intervals for selected haematological parameters: Kenyan infants aged 1 to less than 12 months (current study), compared to published data from Tanzania and United States/Europe.

	Kenya (current study)				*Tanzania (2010)	*US/Europe (2006)
Parameter	Lower 95% reference value	90% Confidence interval for lower reference value	Upper 95% reference value	90% Confidence interval for upper reference value	95% Reference ranges	95% Reference ranges
**Haemoglobin (g/dl)**	7.3	7.0–7.6	13.2	12.9–13.5	8.1–13.2	9.4–13.0
**Haematocrit (%)**	23.5	22.9–24.3	39.2	38.6–39.9	25.1–38.6	28–42
**MCV (fl)**	53.4	52.2–55.0	98.6	97.0–99.3	53.3–96.6	70–98
**Platelets (10**^**3**^**/**μ**L)**	72.7	51.5–89.6	769.2	738.4–822.8	25–708	150–400

*References: Tanzanian data (Buchanan et al. 2010) and USA/European data (Simpkin & Hinchliffe 2006).

**Table 5:** 95% reference intervals for white blood cell counts for Kenyan infants aged 1 to less than 12 months, compared to published data from Tanzania and United States/Europe.

	Kenya (current study)				*Tanzania (2010)	*US/Europe (2006)
Parameter	Lower 95% reference value	90% Confidence interval for lower reference value	Upper 95% reference value	90% Confidence interval for upper reference value	95% Reference ranges	95% Reference ranges
**WBC (x103 cells/μL)**	5.6	5.2–5.9	16.6	16.2–16.9	2.0–17.3	5.0–17.0
**Neutrophils (x103 cells/μL)**	0.7	0.6–0.8	4.1	3.9–4.2	0.7–4.6	0.7–8.0
**Lymphocytes (x103 cells/μL)**	3.3	2.9–3.5	10.2	9.9–10.8	3.3–11.8	3.3–11.5
**Monocytes (x103 cells/μL)**	0.5	0.42–0.53	2.0	1.9–10.8	0.2–1.5	0.2–1.3
**Eosinophils (x103 cells/μL)**	0.06	0.05–0.08	0.9	0.8–1.0	0.1–0.8	0.05–1.1
**Basophils (x103 cells/μL)**	0.01	0.01–0.01	0.07	0.06–0.08	0.01–0.14	0.02–0.13

*References: Tanzanian data (Buchanan et al. 2010) and USA/European data (Simpkin & Hinchliffe 2006).

## Discussion

We have established a set of haematological and biochemistry reference intervals representative of non-underweight children without identified acute or chronic illness aged between 4 weeks to 17 months living in rural Kilifi County. The results describe what is typical in the population, rather than what is optimally healthy.

There was no evidence of gender differences for all haematological and biochemical parameters (see S1, S2 and S3 Tables). This is consistent with previous studies that showed that gender differences for most haematological and chemistry parameters start being evident during adolescence [39–42].

Differences in haematological values among different populations have been attributed to factors such as nutrition, genetic differences, exposure to infectious diseases, environmental factors and socio-economic status. Despite Kenya and Tanzania being neighbouring countries in East Africa, our 2.5^th^ percentile for Hb and Hct; and the 97.5^th^ percentile for WBC, neutrophils, lymphocytes, and basophils were lower than those for Tanzanian children [26]. The 2.5^th^ percentile platelet counts, WBC and monocytes were higher than those for Tanzanian children. The 97.5^th^ percentile platelet counts, monocytes and eosinophils were higher than those for Tanzanian children. Overall, these results suggest that anaemia and inflammation are more common in Kilifi, Kenya than in the Kilimanjaro Region, Tanzania. The 2.5^th^ percentile for neutrophils, lymphocytes, basophils and MCV; and the 97.5^th^ percentile of Hb and Hct were comparable (Table 5 and S4 Table).

Several African studies that have shown lower red blood cell indices in children and adults [16, 24, 25, 43] compared to American or European children [37]; we also observed this for Hb, Hct and MCV(Table 5 and S4 Table). This may be attributed to several factors that are common in the community, including malaria, other parasitic infestations [44, 45], haemoglobinopathies [46] and iron deficiency anaemia [47].

The 2.5^th^ and 97.5^th^ percentile platelet counts in children from Kilifi indicated values that would be classified as thrombocytopenia and thrombocytosis respectively if evaluated by reference ranges among European/American populations. However, they were not associated with clinical features. This was also observed amongst Tanzanian children (Table 5 and S4 Table). Thrombocytopenia can result from impaired platelet production, increased platelet destruction (immune and non–immune) and abnormal vascular distribution e.g. splenic sequestration. One potential cause of thrombocytopenia in African populations is *P*. *falciparum* malaria [48, 49].

Thrombocytosis may be attributed to persistent elevated thrombopoetin levels observed in iron deficiency [50] or inflammation that stimulates its production in the liver [51]. Chronic inflammation is commonly the result of environmental enteric dysfunction, associated with poor sanitation resulting in gut infections, malabsorption and malnutrition [52, 53]. These findings accord with recent studies indicating that platelet counts may be higher in African paediatric populations compared to Caucasian populations [26, 43, 54, 55].

The WBC 2.5^th^ and 97.5^th^ percentiles were higher and lower respectively than those for Tanzanian and American/European populations. Other African studies have shown that the WBC counts in infants tend to be similar or lower than those of Caucasians [26, 43]. The 2.5^th^ percentile for eosinophils was comparable to that of American/European populations while the 97.5^th^ percentile was comparable with for Tanzania (Table 5 and S4 Table).

The 97.5^th^ percentile for neutrophils was higher in the European/American infants compared to our study (Table 5 and S4 Table), as other African studies have demonstrated [26, 43, 56]. This may due to sub-clinical infection. This has also been attributed to a regulatory variant in the Duffy antigen receptor for chemokines [56].

Eosinophils had a lower upper limit in Kilifi when compared to European/American infants, but higher than those for Tanzanian children (Table 5 and S4 Table). Some studies have shown that eosinophils tend to be elevated in African populations due to environmental factors such as helminthiasis [43].

The monocyte 95% intervals were comparable for both Tanzanian and European/American populations, while Kilifi had a higher lower and upper limit (Table 5 and S4 Table). Monocytes have been shown to play an important role in immune mechanisms against protozoal infections such as malaria, with elevated monocytes levels being observed in malaria endemic settings [57–59]. The study area is malaria endemic and this might have contributed to higher monocyte count compared to Tanzanian and European/American infants.

### Strengths and limitations

The major strength of the study is that we analysed a large sample (>1000) of children in a community setting at the time of recruitment for a clinical trial. We believe our reference ranges are representative of typical community children in similar settings in Africa who would ultimately benefit from successful trials. Our study had a number of limitations. Screening could not conclusively rule out all forms of illness such as parasitic infestations, sickle cell anaemia, thalassaemia and micronutrient deficiencies that may have affected the parameters. The HIV status of the children was also not assessed, but in apparently healthy children HIV prevalence would be expected to be less than 1%. The reference ranges are therefore representative of children in the community without acute illness who would typically be eligible for clinical trials, rather than intended to define a state of optimal health. There was no suitable study to compare our biochemistry results with. Because of sample size, biochemistry results were not partitioned by age groups.

## Conclusion

This study, like others in Africa, has shown that commonly used European/American reference ranges do not well suit typical African populations. This reaffirms the need for locally-derived reference values for clinical research in order to appropriately assign eligibility and interpret adverse events.

## Supporting information

S1 Table 95% reference ranges with 90% confidence intervals for selected haematological parameters for Kilifi children aged 1–17 months stratified by gender. (PDF)

S2 Table 95% reference ranges with 90% confidence intervals for selected white blood cells parameters for Kilifi Children aged 1–17 months stratified by gender. (PDF)

S3 Table 95% reference ranges with 90% confidence intervals for selected biochemistry parameters for Kilifi Children aged 1–17 months stratified by gender. (PDF)

S4 Table Selected haematological parameters for Kenyan infants aged 1 to less than 12 months, compared to published data from Tanzania and United States/Europe. (PDF)
