# Supplementary material for: Clinical laboratory reference values amongst children aged 4 weeks to 17 months in Kilifi, Kenya: A cross sectional observational study
Source: PLoS One. 2017 May 11;12(5):e0177382. doi: 10.1371/journal.pone.0177382 (PMC5426761; doi:10.1371/journal.pone.0177382)
Supplement: S1 Table — (PDF) [file pone.0177382.s001.pdf]

Clinical laboratory reference values amongst children aged 4 weeks to 17 months in Kilifi, Kenya: a cross sectional observational study.

Supporting Information: tables

S1 Table: 95% reference ranges with 90% confidence intervals for selected haematological parameters for Kilifi children aged 1-17 months stratified by gender.

	Males			Females			Overall			
**Parameter/Age group**	**N#**	**Median**	**95% Reference ranges**	**N#**	**Median**	**95% Reference values**	**N#**	**Median**	**95% Reference values**	**P-values males vs. females***
**Haemoglobin (g/dl)**										
1-6 months	191	10.2	8.0-14.0	167	10.6	8.3-13.8	358	10.5	8.1-13.8	0.16
6-12 months	187	9.5	7.0-11.5	199	9.7	7.1-11.7	386	9.6	7.0-11.5	0.006
12-17 months	154	9.6	6.9-12.0	152	9.7	7.1-11.9	306	9.6	7.1-11.9	0.31
**Haematocrit (%)**										
1-6 months	160	31.4	24.6-41.9	139	32.3	25.2-42.5	299	31.6	24.8-41.9	0.06
6-12 months	153	29.8	22.6-36.4	167	30.1	23.3-35.9	321	29.9	23.2-36.1	0.05
12-17 months	115	30.6	24.1-36.5	107	30.9	25.4-36.9	222	30.8	25.0-36.6	0.41
**MCHC(g/dL)**										
1-6 months	160	33.0	31.2-34.7	141	33.0	31.0-35.1	301	33.0	31.2-34.7	0.26
6-12 months	156	31.7	29.2-33.8	166	32.1	29.9-33.9	322	31.9	29.5-33.8	0.006
12-17 months	116	31.5	28.9-33.7	108	31.6	28.7-34.0	224	31.6	28.9-33.5	0.48
**MCV(fl)**										
1-6 months	163	80	58-98	142	85	55-102	305	83	57-100	0.20
6-12 months	159	64	51-77	169	66	51-78	328	65	51-78	0.006
12-17 months	118	63	49-79	109	65	50-78	227	64	50-79	0.28
**Platelets (10^3/μL)**										
1-6 months	162	441	93-746	141	444	22-833	303	443	74-765	0.45
6-12 months	152	462	96-804	166	488	155-811	318	471	104-798	0.16
12-17 months	113	483	177-775	104	481	176-783	217	483	184-769	0.68

*p values were assessed using the sum rank test
N# varies for each age group as some lab tests were not done for all participants
