# Supplementary material for: Clinical laboratory reference values amongst children aged 4 weeks to 17 months in Kilifi, Kenya: A cross sectional observational study
Source: PLoS One. 2017 May 11;12(5):e0177382. doi: 10.1371/journal.pone.0177382 (PMC5426761; doi:10.1371/journal.pone.0177382)
Supplement: S2 Table — (PDF) [file pone.0177382.s002.pdf]

Clinical laboratory reference values amongst children aged 4 weeks to 17 months in Kilifi, Kenya: a cross sectional observational study.

Supporting Information: tables

S2 table: 95% reference ranges with 90% confidence intervals for selected white blood cells parameters for Kilifi Children aged 1-17 months stratified by gender

	Males			Females			Overall			
**Parameter/Age group**	**N#**	**Median**	**95% Reference values**	**N#**	**Median**	**95% Reference values**	**N#**	**Median**	**95% Reference values**	**P-value males vs. females***
**WBC(×10³cells/μL)**										
1-6 months	159	8.71	4.60-13.71	134	9.20	5.01-15.93	293	9.10	4.74-14.77	0.01
6-12 months	154	10.70	6.29-17.29	167	10.51	6.90-17.56	321	10.62	6.70-17.39	0.58
12-17 months	113	10.60	5.24-17.08	105	10.91	6.33-16.34	218	10.80	5.84-16.66	0.70
**Neutrophils(×10³ cells/μL)**										
1-6 months	116	1.72	0.66-3.50	95	1.68	0.37-3.73	211	1.71	0.57-3.53	0.78
6-12 months	110	2.36	1.06-4.26	124	2.10	1.02-4.32	234	2.19	1.05-4.25	0.24
12-17 months	67	2.49	0.78-5.55	70	2.27	0.96-5.90	137	2.35	0.95-5.56	0.71
**Lymphocytes(×10³ cells/μL)**										
1-6 months	124	5.31	2.25-8.99	105	5.57	3.39-9.20	229	5.50	3.06-9.04	0.10
6-12 months	112	6.61	3.45-11.31	125	6.49	3.33-10.21	237	6.54	3.38-10.97	0.89
12-17 months	66	6.07	2.62-9.65	71	6.07	3.01-10.08	137	6.07	2.97-9.75	0.65
**Monocytes(×10³ cells/μL)**										
1-6 months	117	0.94	0.37-1.88	101	1.04	0.35-1.91	218	0.96	0.38-1.89	0.24
6-12 months	107	1.11	0.62-2.17	127	1.10	0.53-2.04	234	1.11	0.60-2.06	0.51
12-17 months	65	1.01	0.49-1.79	69	1.07	0.43-1.92	134	1.03	0.48-1.91	0.88
**Eosinophils(×10³ cells/μL)**										
1-6 months	112	0.27	0.10-0.59	99	0.29	0.05-0.75	211	0.28	0.07-0.70	0.71
6-12 months	109	0.38	0.04-1.23	123	0.37	0.09-1.20	232	0.38	0.07-1.20	0.56
12-17 months	64	0.43	0.05-1.25	70	0.44	0.04-1.16	134	0.44	0.05-1.20	0.76

**Basophils(×10^3 cells/µL)**										
1-6 months	120	0.02	0.01-0.07	94	0.02	0.00-0.06	214	0.02	0.01-0.06	0.26
6-12 months	106	0.02	0.01-0.04	115	0.02	0.01-0.04	221	0.02	0.01-0.04	0.59
12-17 months	70	0.02	0.01-0.05	78	0.02	0.01-0.06	148	0.02	0.01-0.06	0.28

*p values were assessed using the sum rank test

N# varies for each age group as some lab tests were not done for all participants
