# Supplementary material for: Clinical laboratory reference values amongst children aged 4 weeks to 17 months in Kilifi, Kenya: A cross sectional observational study
Source: PLoS One. 2017 May 11;12(5):e0177382. doi: 10.1371/journal.pone.0177382 (PMC5426761; doi:10.1371/journal.pone.0177382)
Supplement: S3 Table — (PDF) [file pone.0177382.s003.pdf]

Clinical laboratory reference values amongst children aged 4 weeks to 17 months in Kilifi, Kenya: a cross sectional observational study.

Supporting Information: tables

S3 table: 95% reference ranges with 90% confidence intervals for selected biochemistry parameters for Kilifi Children aged 1-17 months stratified by gender.

	Males			Females			Overall			
**Parameter**	**N#**	**Median**	**95% Reference values**	**N#**	**Median**	**95% Reference values**	**N#**	**Median**	**95% Reference values**	**P-values males vs. females***
**Creatinine(µmol/L)**	208	36	27-45	211	36	26-45	419	36	27-45	0.73
**ALT(IU/L)**	203	19	8-34	205	19	10-33	408	19	9-34	0.99

*p values were assessed using the sum rank test
N# varies for each age group as some lab tests were not done for all participants
