# Supplementary material for: Clinical laboratory reference values amongst children aged 4 weeks to 17 months in Kilifi, Kenya: A cross sectional observational study
Source: PLoS One. 2017 May 11;12(5):e0177382. doi: 10.1371/journal.pone.0177382 (PMC5426761; doi:10.1371/journal.pone.0177382)
Supplement: S4 Table — (PDF) [file pone.0177382.s004.pdf]

Supplementary Table 4: Selected haematological parameters for Kenyan infants aged 1 to less than 12 months, compared to published data from Tanzania and United States/Europe.

	Kenya Lower 95% reference value	Tanzania lower 95% reference value*	P-value (t-test)	United states/Europe lower 95% reference value*	P-value (t-test)	Kenya upper 95% reference value*	Tanzania Upper 95% reference value*	P-value (t-test)	United states/Europe upper 95% reference value*	P-value (t-test)
**Haematology**										
**Haemoglobin (g/dl)**	7.3	8.1	<0.001	9.4	<0.001	13.2	13.2	1.0	13.0	0.27
**Haematocrit (%)**	23.5	25.1	0.002	28	<0.001	39.2	39.9	0.13	42	<0.001
**MCV (fl)**	53.4	53.3	0.91	70	<0.001	98.6	96.6	0.004	98	0.39
**Platelets (10^3/µL)**	72.7	25	<0.001	150	<0.001	769.2	708	0.02	400	<0.001
**White blood cell counts**										
**WBC (x10^3 cells/µL)**	5.6	2.0	<0.001	5.0	0.001	16.6	17.3	0.001	17.0	0.06
**Neutrophils (x10^3 cells/µL)**	0.7	0.7	1.0	0.7	1.0	4.1	4.6	<0.001	8.0	<0.001
**Lymphocytes (x10^3 cells/µL)**	3.3	3.3	1.0	3.3	1.0	10.2	11.8	<0.001	11.5	<0.001
**Monocytes (x10^3 cells/µL)**	0.5	0.2	<0.001	0.2	<0.001	2.0	1.5	<0.001	1.3	<0.001
**Eosinophils (x10^3 cells/µL)**	0.06	0.1	<0.001	0.05	1.0	0.9	0.8	<0.001	1.1	<0.001
**Basophils (x10^3 cells/µL)**	0.01	0.01	1.0	0.02	<0.001	0.07	0.14	<0.001	0.13	<0.001

*References: Tanzanian data (Buchanan et al. 2010) and USA/European data (Simpkin & Hinchliffe 2006) and Kenya values are from the current study
